# Supplementary material for: Tandem Repeat DNA Provides Many Cytological Markers for Hybrid Zone Analysis in Two Subspecies of the Grasshopper Chorthippus parallelus
Source: Genes (Basel). 2023 Feb 3;14(2):397. doi: 10.3390/genes14020397 (PMC9957195; doi:10.3390/genes14020397)
Supplement: Supplementary file 1 [file genes-14-00397-s001.zip › genes-2164885-supplementary.pdf]

Table S1. Primers designed to amplify 82 TR families.

TR family	Forward	Reverse	PCR amplification
CpaTR001-148	TGGTTGGAATTAAAAAGATACGTGT	GAATCCAAATACCAATTTTTGTAGTTC	Yes
CpaTR002-188	CGAGGGAGGACTCGAACCTC	GGGCATGGGTGTTTGTGTTG	No
CpaTR003-133	ACATCGAGAACCTGTCAACCA	TGTACCCTTGTCCGCTCAGT	Yes
CpaTR004-335	TTGTGACGTTTGATTTTTATATAGGTA	GTGAACTCTGAAACTACCCAAC	Yes
CpaTR005-130	AGTCCGTAGGTAAAGTACCCTC	CTTGACGAAATTTTGCACACTTGAC	Yes
CpaTR006-11	TCGCGCAGACTTCGCGCA	GCGCGAAGTCTGCGCGAA	Yes
CpaTR007-21	CTGGGTGCAGGCCTAGGTTA	CTAGGCCTGCACCCAGGTAA	Yes
CpaTR008-331	GTTACTGAAAACATTTAAAATTGCGA	ACTGTCGTTCATCTATTTGAAACC	Yes
CpaTR009-172	TGCCCCAAACAATGCTCAAGA	AGGCATGATTTCATCAAAATCGGAC	Yes
CpaTR010-275	TACTGCACGACTGACAGTGG	AGGGCAATGGTCTCTGCTG	Yes
CpaTR011-213	TGTGAAGAAATTGTTACTTCTGTATCA	CTTGCTGCAGATACACTCCTGGG	Yes
CpaTR012-247	CCACCGTGACACAGTACGTAC	TGGAGGCATAGGTGCCACTG	Yes
CpaTR013-55	GCTATCGAACATATAAAAACGCAC	TTGGTTAGGTATAGGTTACCTTAGG	Yes
CpaTR014-299	CTCTCACAGGACGCAGTGCC	GAGACTGCATTACCCACACAATCTT	Yes
CpaTR015-284	CAATCCGAACCCCAGGAACC	TGCCCTAATGACGCTGCAAA	Yes
CpaTR016-7	TGAGATATGAGATATGAGATATGAG	CATATCTCATATCTCATATCTCATA	Yes
CpaTR017-289	CCGACTGGGGACAAGTGGTA	GGAAACCGAACCAAGGACCC	Yes
CpaTR018-166	TGAACAACGACAGACCCACATTTA	CACTTCAATCTCCAAAACTACTTC	Yes
CpaTR020-246	TTGGGCGCCAGCTTTAGAAG	AACGTAAATAAACAATAAGCCTGG	Yes
CpaTR021-89	CCGTCTAGTGCAGCGTCAC	TCGTCTCAGCTGCTTGATGC	Yes
CpaTR022-239	GGCACCACGTCTGCAGTAAA	GACCTACCAAGGCCTCGTTG	Yes
CpaTR023-176	CCTTGCCCTCCAACTTCAGG	TGTCCAGTATTCAGTTGTCCA	Yes
CpaTR024-210	AGAGAGGGAAGGGTGAGCTAT	CAGAGCTCCAGACTGTCAGCAG	Yes
CpaTR025-248	TGTGCTGTGTTCAAAAGTCAAAG	GCTTGTAGCCAGTGTAGTGTGC	Yes
CpaTR026-239	GGGAAATGTGTTAGCCCTTCCAGT	AGAGCACACTGAGTGCATACGT	Yes
CpaTR028-148	GGTGGAGTTTGAAGAGTTATGCG	GGAAATACCAATCCCTGCAACTG	Yes
CpaTR029-290	TCACCCATTAAACGTGTTCGA	ACAAAGAACTACAAACGAAAATTCGT	Yes
CpaTR030-79	ACTACCAGTCCTGTGTGAC	CAATGGCTGTGGTAGCAGCA	Yes
CpaTR031-236	TCAGTTGGTCGCCAGCCTTT	TGATAACGTAAATAAACAAACGG	Yes
CpaTR032-20	AACCCAATGTACCTTAACCTAACC	GTTAGGTTAAGGTACATTGGGTTA	Yes
CpaTR033-222	GCTGTAGGCTGACAAGTACACAC	AGCAGCAGTGCAGTAGAAGG	Yes
CpaTR034-61	GCAATCACTCTTGCATCTCA	TGCTTGTGGGAGTGAGTGTTC	Yes
CpaTR035-165	TTTCCACACCACAGTCCCCT	ACTGTTGTTGTCTGCTGTGGT	Yes
CpaTR036-168	ATGGAAACGACTTATTGTCATTTC	TGGAAATGCACAAAACACGA	Yes
CpaTR037-147	GCGAATATGACGAGAATATTGTATATG	GCGAAAACAGCTTAAACAGGGT	Yes
CpaTR038-203	ACGGCTCCGACGCATCAAGC	AGGGGAGGCCGAGGTGTGTG	Yes
CpaTR039-139	GGGCTGGTCCCCCTACACCT	GCGGAGTAGGCTACCTGACTGGAG	Yes
CpaTR040-161	ACAGCAAAGTCCCACACAAT	TAACGGTCTTGCTTGGGCAA	Yes
CpaTR042-151	GCACCGTCTGATCCAAGTGT	ACACACGCTTGACTCACCAC	Yes
CpaTR043-237	ATATAACGATAAATAAACAACTCTTCA	TATGTGTTTTGTGATTTAGTTTGCT	Yes
CpaTR044-205	GTCTTCCCATGGTGTGAGTTGT	ACTGGAAATTCGAAAAGGGATGCT	Yes
CpaTR046-157	AAGAAAGAGCAAGTTACGAGAAT	ACTTCCATGCTGTTACACTAACG	Yes
CpaTR047-287	ACCTGGCTTGGCGTTGAAAT	TTCGTCGCAGCTCTTCAACA	Yes
CpaTR048-15	TGCCCTGAAAGTGGGTGCCC	GGCACCCACTTTCAGGGCAC	Yes
CpaTR049-215	CGGCGCTCCTAATCACGAGCC	GCTGTCGCTCTTGCTGCGCT	Yes
CpaTR050-288	GGAACCCACACCATGAACCA	TGTGTGAGACCACAGTTTCA	Yes
CpaTR051-53	CCAGTGACATGAAATTTGTTTATAAGC	GGTAAAATGTTATAAACGAGAACTGT	Yes
CpaTR052-43	CAGTAATCTGTAGAAATATCAGGGTAC	GTATATTGCCACTATTTGTACCCTG	No
CpaTR053-405	ACTGATAAATCAAGATACGTGACACAA	TGAGTGTTTGCGTGCATATT	Yes
CpaTR057-102	TACTGGGAGCTGGGCACGGG	ACCAGCACCCCTCCACACTGT	Yes

CpaTR058-196	CCTGTGACGCCTCCCCTC	GCTCAACAAAAGAAAAGCAACATTCTG	Yes
CpaTR060-14	TGGAGGTAGCAATTTGGAGG	TGGAGGTAGCAATTTGGAGG	No
CpaTR061-27	ACAGAGACAGGGGAGAGGAGGA	CTCCCCTGTCTCTGTTCACTTC	Yes
CpaTR062-56	GCAGGGATGAGTTGTGCCTGAGT	TGCCATGGGCTTCGTCACTCA	Yes
CpaTR063-92	CTGATGTGGCGACGGGCTGG	CCACGCCTCCTTCACGTGCC	Yes
CpaTR065-379	TGGCCGGTTATCAGGGCCGA	CCACTTGCCCAGCACTTGCCA	Yes
CpaTR068-155	AGAGAGGAGAAGGCCAGCTG	TCAACGTGGGAGGCGTCTAT	Yes
CpaTR069-89	TGTCCCACAAATTGGCTGTGA	TCCTGTTGGAAACTCCTCTCCA	Yes
CpaTR070-84	TGTGGGGCCTTGTTCCAGTGTGTAT	TGGCAAATCTTTTACTGAGTTGGGTAC	Yes
CpaTR071-164	CGGTTACACGCGAATTTTCA	CGACACCATGTTGGTAATGGT	Yes
CpaTR072-144	TCCCTTTACACAAAAATTCCCCCA	ACTTTATGAAAAATCGAAAGCAAAGGA	Yes
CpaTR073-67	CCAAAGGGCAGAGTCAGTCC	TGGTCAGCCTAGCTGTGTTG	Yes
CpaTR074-186	TAGCATGTGATGGTGTGGGG	TCAGTATTGGCTTTTCCTCAGT	Yes
CpaTR075-45	GCTAGTAAAGGGCCATGCTGCA	GCTGTGTGACATGCTCTCGTCA	Yes
CpaTR076-168	AAAGCATGGTCACGTCCGAC	ACACATTTCCCCACACAGCTAAAA	Yes
CpaTR077-16	TGCGAGTGCACACCTGTGCG	ACAGGTGTGCACTCGCACAG	Yes
CpaTR081-170	AAGAAACAACGATTAATTTCAGAATTA	ACGGTTGCAACTGGTCATATTGAG	Yes
CpaTR085-37	AGATAAGTGGGATCACATTCCCC	AAAACCTGGAAGGGGGAATG	No
CpaTR087-247	AACGTCTTTGGAAGCACTATAGT	AGCAAACAAAAACTACTTCTGTACAAC	Yes
CpaTR091-49	CCCTCCTATTGCAGTCTCAT	GGGAGATGTGATGACACAGACTGA	Yes
CpaTR094-170	ACAAAGAAACACTGATTAAATTGAAGT	CGGTCATAACTGGTCTGGTTGAA	Yes
CpaTR097-108	AGGGTAAATCCAGTAATGCTCCA	AACCTACGTAAACAACATGAAAATT	Yes
CpaTR098-169	TGAGCTACCGTCCTTGCACT	AGAGAGTAAACTGGAGTCAGGACG	No
CpaTR100-295	GGGATGCAGGTGGTCAGGACG	CCCCACCCCCAGTATATGAT	Yes
CpaTR101-112	AGTCCTAGCTCCACCACACGT	ACATCCCATACGTACACTGCA	Yes
CpaTR102-127	GGTAGATAGCCATAGAGGCGATGG	ACCTGCATAGTGTTTTGCCCTTCA	Yes
CpaTR103-33	TCCCCACGTCCTCTAGTGCCA	GTGGGGACAGATGATGAGATG	Yes
CpaTR104-269	ACGCTTCCAAACGAACTCAAAGACA	TAGCTGCCAAGGCTTGACGGT	Yes
CpaTR107-237	TGGGGAGGCAGGTGGTCAGG	TCCTGTACGTAATTTACACTCCTG	Yes
CpaTR108-37	GCCTCCCTACCTGTGACCATGAC	GGCTACAACTGTACAATGTCATGGT	Yes
CpaTR109-108	GGCACGGTAGATTCCGACAAGA	GCCGCAGGACGATCAGGAGA	Yes
CpaTR110-159	TCACAATCACGTCCAGGGGGTG	ACCCCTATCACTTACACTGC	Yes

Table S2. Sequence characteristics of the 110 TR families found in the genome of *C. parallelus*. RUL= Repeat Unit Length, A+T= adenin+thymine content, SF= superfamily, Subfam= number of subfamilies, CPPabun and CPE abun= genomic abundance per subspecies. CPP= *Chorthippus parallelus parallelus*, CPE= *Chorthippus parallelus erythropus*. FISH patterns: B= banded, DB= dotted-banded, D= dotted, NS= no signal.

					Genomic abundance (%)		FISH pattern		
TR_name	RUL	A+T (%)	SF	Subfam	CPPabun	CPEabun	CPP	CPE	TE homology
CpaTR001-148	148	66.89	1	3	0.91623	1.00776	NS	NS	Helitron-N10_LMi
CpaTR002-188	188	49.47		1	0.52640	0.45403			hAT-11_LMi, DNA8-16_LMi
CpaTR003-133	133	63.91		1	0.28719	0.27922	NS	NS	DNA8-5_LMi, Mariner-N21_LMi, DNA8-11B_LMi
CpaTR004-335	335	61.19		2	0.27214	0.23718	B	B	Helitron-N19B_LMi, DNA-7_LMi
CpaTR005-130	130	62.31		2	0.24854	0.24244	NS	NS	DNA8-16B_LMi, hAT-N12_LMi
CpaTR006-11	11	36.36		2	0.21965	0.14931	B	B	
CpaTR007-21	21	38.10		1	0.09553	0.10323	B	B	
CpaTR008-331	331	64.05	2	1	0.09247	0.09413	B	B	Helitron-N11B_LMi
CpaTR009-172	172	63.95	2	2	0.07876	0.08039	B	B	Helitron-N11B_LMi
CpaTR010-275	275	52.00		2	0.07597	0.08906	DB	DB	
CpaTR011-213	213	58.69		2	0.06702	0.06434	B	B	
CpaTR012-247	247	53.85		1	0.05453	0.06219	B	B	hAT-30N1_LMi
CpaTR013-55	55	60.00		3	0.05330	0.05227	B	NS	
CpaTR014-299	299	64.21		2	0.03948	0.07974	D	D	DNA8-9_LMi
CpaTR015-284	284	65.14		1	0.03656	0.04484	NS	NS	
CpaTR016-7	7	71.43		1	0.03373	0.00464	B	B	
CpaTR017-289	289	51.90		2	0.03343	0.03444	B	B	
CpaTR018-166	166	62.65	2	1	0.02823	0.02842	NS	NS	Helitron-N11B_LMi
CpaTR019-5-tel	5	60.00		1	0.02449	0.02959	B	B	
CpaTR020-246	246	59.35	4	2	0.02242	0.02403	B	B	CR1-1_LMi
CpaTR021-89	89	41.57		2	0.02069	0.01463	D	NS	
CpaTR022-239	239	50.21		1	0.02035	0.01544	NS	NS	
CpaTR023-176	176	57.95		1	0.02004	0.03260	D	D	
CpaTR024-210	210	61.43	3	1	0.01966	0.02190	B	B	
CpaTR025-248	248	62.90	5	2	0.01960	0.02061	B	NS	
CpaTR026-239	239	59.83	3	1	0.01916	0.04248	B	B	
CpaTR027-82	82	47.56		1	0.01660	0.00811			
CpaTR028-148	148	59.46	1	2	0.01586	0.01787	B	B	Helitron-N10_LMi
CpaTR029-290	290	65.52		1	0.01523	0.01715	NS	D	
CpaTR030-79	79	45.57		3	0.01368	0.01075	B	B	
CpaTR031-236	236	59.75	4	2	0.01193	0.00776	D	D	CR1-1_LMi
CpaTR032-20	20	65.00		2	0.01080	0.02159	B	B	
CpaTR033-222	222	63.06	5	2	0.01029	0.01255	NS	B	
CpaTR034-61	61	57.38		1	0.00963	0.01749	NS	B	
CpaTR035-165	165	57.58		1	0.00941	0.01044	NS	NS	
CpaTR036-168	168	58.93		3	0.00923	0.00433	B	B	PST1 (Satellite)
CpaTR037-147	147	63.95		2	0.00912	0.00911	NS	NS	
CpaTR038-203	203	35.96		1	0.00876	0.00853	D	D	
CpaTR039-139	139	46.04		2	0.00817	0.00803	B	B	
CpaTR040-161	161	58.39	6	3	0.00798	0.00494	B	B	
CpaTR041-388	388	54.64		1	0.00772	0.00606			
CpaTR042-151	151	50.99		1	0.00771	0.00713	NS	NS	Mariner-73_LMi
CpaTR043-237	237	61.18	5	3	0.00735	0.00552	NS	D	
CpaTR044-205	205	59.02		1	0.00734	0.00893	B	B	
CpaTR045-220	220	62.73	10	1	0.00731	0.01056			CR1-4_LMi
CpaTR046-157	157	68.15	7	2	0.00730	0.01031	B	B	

CpaTR047-287	287	62.37		1	0.00655	0.01115	NS	B	
CpaTR048-15	15	40.00		1	0.00639	0.00453	B	B	
CpaTR049-215	215	59.53		1	0.00625	0.00196	B	B	
CpaTR050-288	288	67.01		1	0.00584	0.00830	NS	B	RTE-53_LMi
CpaTR051-53	53	69.81		1	0.00566	0.01019	NS	NS	
CpaTR052-43	43	67.44		1	0.00549	0.00361			
CpaTR053-405	405	60.74	9	1	0.00536	0.00835	B	NS	CR1-2_LMi
CpaTR054-180	180	59.44		1	0.00533	0.00256			
CpaTR055-286	286	50.70	11	1	0.00519	0.00474			
CpaTR056-206	206	62.62		1	0.00509	0.00451			P-2N1_LMi
CpaTR057-102	102	39.22		1	0.00493	0.00246	NS	B	
CpaTR058-196	196	60.20		2	0.00481	0.00475	B	NS	
CpaTR059-54	54	53.70		1	0.00477	0.00743			
CpaTR060-14	14	57.14		1	0.00469	0.00202			
CpaTR061-27	27	44.44		1	0.00449	0.00507	B	B	
CpaTR062-56	56	46.43		1	0.00443	0.00442	B	B	hAT-N34_LMi
CpaTR063-92	92	39.13		1	0.00441	0.00235	B	B	
CpaTR064-51	51	41.18		1	0.00441	0.00216			
CpaTR065-379	379	63.32		1	0.00422	0.00264	B	B	Penelope-6_LMi
CpaTR066-247	247	59.51	4	2	0.00419	0.00339			
CpaTR067-177	177	60.45		2	0.00408	0.00465			
CpaTR068-155	155	61.29		1	0.00402	0.00225	B	B	
CpaTR069-89	89	63.27		1	0.00398	0.00133	B	B	
CpaTR070-84	84	58.33	8	1	0.00392	0.00385	NS	NS	
CpaTR071-164	164	68.29		1	0.00369	0.00694	NS	NS	
CpaTR072-144	144	76.39		1	0.00361	0.00508	NS	NS	
CpaTR073-67	67	52.24		1	0.00359	0.00378	NS	NS	
CpaTR074-186	186	57.53		1	0.00351	0.00795	B	B	
CpaTR075-45	45	48.89		1	0.00342	0.00226	B	B	
CpaTR076-168	168	63.10		2	0.00336	0.00470	NS	B	
CpaTR077-16	16	37.50		1	0.00323	0.00839	B	B	
CpaTR078-61	61	54.10		2	0.00322	0.00568			
CpaTR079-262	262	47.71		1	0.00313	0.00314			
CpaTR080-258	258	58.53	9	1	0.00310	0.00396			CR1-2_LMi
CpaTR081-170	170	63.53	6	2	0.00300	0.00083	NS	NS	
CpaTR082-158	158	68.35	7	1	0.00281	0.00416			
CpaTR083-84	84	59.52	8	1	0.00267	0.00384			
CpaTR084-183	183	60.66		2	0.00264	0.00410			
CpaTR085-37	37	56.76		1	0.00255	0.00492			
CpaTR086-227	227	59.91	10	2	0.00240	0.00386			
CpaTR087-247	247	61.54	4	2	0.00226	0.00225	NS	NS	
CpaTR088-170	170	62.35	6	2	0.00186	0.00302			
CpaTR089-112	112	46.88	11	1	0.00184	0.00204			
CpaTR090-206	206	71.81		1	0.00166	0.00374			
CpaTR091-49	49	57.14		1	0.00137	0.00187	B	B	
CpaTR092-99	99	57.58		1	0.00131	0.00046			
CpaTR093-142	142	62.68	12	1	0.00122	0.00057			
CpaTR094-170	170	63.53	6	1	0.00116	0.00144	NS	B	
CpaTR095-56	56	60.71		1	0.00098	0.00032			
CpaTR096-70	70	58.57		1	0.00071	0.00150			
CpaTR097-108	108	69.44		2	0.00066	0.00409	NS	B	
CpaTR098-169	169	62.72		1	0.00060	0.00366			
CpaTR099-82	82	59.76	12	1	0.00056	0.00059			
CpaTR100-295	295	51.19	13	1	0.00054	0.00175	NS	B	

CpaTR101-112	112	58.04		1	0.00051	0.00077	NS	NS
CpaTR102-127	127	50.39	11	1	0.00042	0.00025	NS	NS
CpaTR103-33	33	45.45		1	0.00042	0.00544	NS	B
CpaTR104-269	269	62.45		1	0.00040	0.02635	B	B
CpaTR105-6	6	66.67		1	0.00021	0.00011		
CpaTR106-96	96	48.96	11	1	0.00019	0.00017		
CpaTR107-237	237	49.79	13	1	0.00006	0.00088	NS	B
CpaTR108-37	37	51.35		2	0.00003	0.00382	NS	NS
CpaTR109-108	108	54.63		1	0.00003	0.00058	NS	NS
CpaTR110-159	159	51.57	13	1	0.00003	0.00028	B	B

Table S3. Molecular properties of the 50 TR families showing FISH bands in at least one of the subspecies. RUL= Repeat Unit Length, A+T= adenin+thymine content, SF= superfamily, Subfam= number of subfamilies, CPPabun and CPE abun= genomic abundance per subspecies, CPPdiv and CPEdiv= K2P divergence, CPP_TSI and CPE_TSI= tandem structure index in each subspecies, CPP_RPS and CPE_RPS= relative peak size index for homogenization, CPP_DIVPEAK and CPE_DIVPEAK= divergence corresponding to the highest abundance peak in the RL, as an index of TR degeneration. TE_homology= TRs displaying homology, in their consensus sequence, with transposable elements (TEs) in RepBase.

					Genomic abundance (%)		Divergence (%)		Tandem structure index		Homogenization coefficient (RPS) (%)		Degeneration coefficient (DIVPEAK)		
Satellite	RUL	A+T (%)	SF	Subfam	CPPabun	CPEabun	CPPdiv	CPEdiv	CPP_TSI	CPE_TSI	CPP_RPS	CPE_RPS	CPP_DIVPEAK	CPE_DIVPEAK	TE_homology
CpaTR004-335	335	61.19		2	0.27214	0.23718	5.54	5.62	0.96	0.9630	58.2	57.8	0.5	0.5	Helitron-N19B_LMi, DNA-7_LMi
CpaTR006-11	11	36.36		2	0.21965	0.14931	4.01	3.89	1.00	0.9955	93.3	93.5	4.0	4.0	
CpaTR007-21	21	38.10		1	0.09553	0.10323	5.80	5.79	0.95	0.9546	80.5	80.9	5.0	5.0	
CpaTR008-331	331	64.05	2	1	0.09247	0.09413	8.71	8.58	0.88	0.8753	45.0	46.7	3.0	4.0	Helitron-N11B_LMi
CpaTR009-172	172	63.95	2	2	0.07876	0.08039	13.80	13.82	0.82	0.8239	19.3	17.7	26.0	4.5	Helitron-N11B_LMi
CpaTR010-275	275	52.00		2	0.07597	0.08906	8.87	8.65	0.99	0.9881	34.2	36.3	5.0	5.0	
CpaTR011-213	213	58.69		2	0.06702	0.06434	3.99	3.92	0.98	0.9810	75.9	76.6	2.5	3.0	
CpaTR012-247	247	53.85		1	0.05453	0.06219	3.02	2.81	0.88	0.9047	88.4	90.3	2.0	2.0	hAT-30N1_LMi
CpaTR013-55	55	60.00		3	0.05330	0.05227	7.99	8.06	0.70	0.7003	45.8	46.2	5.5	6.5	
CpaTR016-7	7	71.43		1	0.03373	0.00464	3.43	3.99	1.00	0.9976	72.8	58.7	2.5	5.0	
CpaTR017-289	289	51.90		2	0.03343	0.03444	17.25	17.52	0.92	0.9127	39.3	34.6	2.0	1.5	
CpaTR020-246	246	59.35	4	2	0.02242	0.02403	4.73	4.78	0.95	0.9508	58.4	49.7	2.0	1.5	CR1-1_LMi
CpaTR024-210	210	61.43	3	1	0.01966	0.02190	3.81	3.40	1.00	0.9978	80.1	84.1	2.5	3.0	
CpaTR025-248	248	62.90	5	2	0.01960	0.02061	4.94	4.38	0.91	0.9208	48.8	50.2	0.5	0.5	
CpaTR026-239	239	59.83	3	1	0.01916	0.04248	4.30	2.90	1.00	0.9975	57.5	82.3	0.5	2.5	
CpaTR028-148	148	59.46	1	2	0.01586	0.01787	9.27	9.29	0.95	0.9465	64.1	61.8	4.0	3.5	Helitron-N10_LMi
CpaTR030-79	79	45.57		3	0.01368	0.01075	5.82	6.27	0.99	0.9894	70.4	67.6	5.0	4.5	
CpaTR032-20	20	65.00		2	0.01080	0.02159	7.15	7.05	0.93	0.9541	56.9	71.6	5.0	6.0	
CpaTR033-222	222	63.06	5	2	0.01029	0.01255	3.43	3.10	0.92	0.9305	79.1	85.8	1.5	1.5	
CpaTR034-61	61	57.38		1	0.00963	0.01749	13.04	9.77	0.03	0.8171	52.5	68.8	15.0	2.0	
CpaTR036-168	168	58.93		3	0.00923	0.00433	6.83	6.53	0.95	0.9506	62.3	54.8	8.0	8.0	PST1
CpaTR039-139	139	46.04		2	0.00817	0.00803	3.88	3.68	1.00	0.9963	78.8	78.6	1.5	1.5	
CpaTR040-161	161	58.39	6	3	0.00798	0.00494	7.40	8.40	0.98	0.9622	62.5	49.9	1.5	2.0	
CpaTR044-205	205	59.02		1	0.00734	0.00893	5.71	5.69	1.00	0.9966	72.8	77.0	4.5	5.0	
CpaTR046-157	157	68.15	7	2	0.00730	0.01031	7.63	7.40	0.95	0.9512	47.7	52.0	3.5	3.5	
CpaTR047-287	287	62.37		1	0.00655	0.01115	10.62	7.21	0.50	0.7383	26.5	56.9	0.0	0.0	
CpaTR048-15	15	40.00		1	0.00639	0.00453	11.67	12.56	0.99	0.9862	61.2	53.3	10.0	10.0	
CpaTR049-215	215	59.53		1	0.00625	0.00196	4.32	3.46	0.98	0.9775	79.3	80.1	3.0	1.0	
CpaTR050-288	288	67.01		1	0.00584	0.00830	3.26	2.96	0.01	0.2381	64.2	73.4	0.0	0.0	RTE-53_LMi
CpaTR053-405	405	60.74	9	1	0.00536	0.00835	6.83	4.35	0.87	0.9150	43.2	66.2	1.5	0.5	CR1-2_LMi
CpaTR057-102	102	39.22		1	0.00493	0.00246	18.23	17.49	0.97	0.9711	46.7	47.9	19.5	20.5	
CpaTR058-196	196	60.20		2	0.00481	0.00475	9.31	9.93	0.93	0.9081	44.1	38.8	3.5	1.5	
CpaTR061-27	27	44.44		1	0.00449	0.00507	9.72	10.22	1.00	0.9972	50.5	45.1	5.0	6.5	
CpaTR062-56	56	46.43		1	0.00443	0.00442	7.73	6.93	0.93	0.9449	78.8	84.9	2.5	3.0	hAT-N34_LMi
CpaTR063-92	92	39.13		1	0.00441	0.00235	6.28	5.22	0.99	0.9897	71.1	65.9	4.5	5.0	
CpaTR065-379	379	63.32		1	0.00422	0.00264	9.02	11.49	0.72	0.5501	50.7	22.9	0.0	0.5	Penelope-6_LMi
CpaTR068-155	155	61.29		1	0.00402	0.00225	3.56	2.10	1.00	0.9989	69.0	94.5	3.0	1.5	
CpaTR069-89	89	63.27		1	0.00398	0.00133	9.48	9.97	0.97	0.9285	64.9	54.9	4.5	3.5	
CpaTR074-186	186	57.53		1	0.00351	0.00795	7.59	6.28	0.99	0.9897	65.4	66.0	6.0	5.0	
CpaTR075-45	45	48.89		1	0.00342	0.00226	4.36	4.96	0.99	0.9960	76.1	62.3	2.5	3.5	
CpaTR076-168	168	63.10		2	0.00336	0.00470	6.80	6.56	0.89	0.8824	57.4	56.8	3.5	4.0	
CpaTR077-16	16	37.50		1	0.00323	0.00839	8.55	8.66	0.99	0.9907	66.6	61.0	7.5	8.5	
CpaTR091-49	49	57.14		1	0.00137	0.00187	3.59	2.49	0.97	0.9894	92.9	95.5	2.0	1.5	
CpaTR094-170	170	63.53	6	1	0.00116	0.00144	14.64	15.27	0.87	0.8857	41.0	40.8	14.5	16.5	
CpaTR097-108	108	69.44		2	0.00066	0.00409	16.77	6.99	0.56	0.9278	45.5	76.9	22.5	3.0	
CpaTR100-295	295	51.19	13	1	0.00054	0.00175	3.44	2.32	0.92	0.9795	72.1	89.8	1.5	1.0	

CpaTR103-33	33	45.45		1	0.00042	0.00544	18.27	5.34	0.11	0.9918	46.5	86.6	16.5	3.5
CpaTR104-269	269	62.45		1	0.00040	0.02635	23.43	3.54	0.82	0.9967	32.4	84.7	22.0	2.0
CpaTR107-237	237	49.79	13	1	0.00006	0.00088	6.62	2.10	0.88	0.9855	52.0	94.5	11.0	1.0
CpaTR110-159	159	51.57	13	1	0.00003	0.00028	3.13	12.28	0.85	0.9395	55.5	93.5	6.0	0.0
